# Manganese(III) Porphyrin-based Potentiometric Sensors for Diclofenac Assay in Pharmaceutical Preparations

**DOI:** 10.3390/s101008850

**Published:** 2010-09-28

**Authors:** Dana Vlascici, Stela Pruneanu, Liliana Olenic, Florina Pogacean, Vasile Ostafe, Vlad Chiriac, Elena Maria Pica, Liviu Calin Bolundut, Luminita Nica, Eugenia Fagadar-Cosma

**Affiliations:** 1 West University of Timisoara, 4 V. Parvan Ave, Timisoara 300223 Timis, Romania; E-Mails: vostafe@yahoo.com (V.O.); chiriac@mail.dnttm.ro (V.C.); 2 National Institute of Research and Development for Isotopic and Molecular Technologies, 65-103 Donath Street 400293, Cluj-Napoca, Romania; E-Mails: stela.pruneanu@itim-cj.ro (S.T.); liliana.olenic@itim-cj.ro (L.O.); florina.pogacean@itim-cj.ro (F.P.); 3 Technical University of Cluj-Napoca, Faculty of Science and Materials Engineering, 28 Memorandumului Street, 400114, Cluj-Napoca, Romania; E-Mails: empica@yahoo.com (E.M.P.); liviu.bolundut@chem.utcluj.ro (L.C.B.); 4 Victor Babes University of Medicine and Pharmacy Timisoara, Revolutiei Ave. No. 9, Timisoara, Romania; E-Mail: purcro@yahoo.com; 5 Institute of Chemistry—Timişoara of Romanian Academy, 24 M. Viteazul Ave, 300223-Timisoara, Romania; E-Mail; efagadar@yahoo.com

**Keywords:** diclofenac, manganese porphyrins, ion–selective electrodes, potentiometry, electrochemical impedance spectroscopy, pharmaceuticals

## Abstract

Two manganese(III) porphyrins: manganese(III) tetraphenylporphyrin chloride and manganese(III)-tetrakis(3-hydroxyphenyl)porphyrin chloride were tested as ionophores for the construction of new diclofenac–selective electrodes. The electroactive material was incorporated either in PVC or a sol–gel matrix. The effect of different plasticizers and additives (anionic and cationic) on the potentiometric response was studied. The best results were obtained for the PVC membrane plasticized with dioctylphtalate and having sodium tetraphenylborate as a lipophilic anionic additive incorporated. The sensor response was linear in the concentration range 3 × 10^−6^ – 1 × 10^−2^ M with a slope of −59.7 mV/dec diclofenac, a detection limit of 1.5 × 10^−6^ M and very good selectivity coefficients. It was used for the determination of diclofenac in pharmaceutical preparations, by direct potentiometry. The results were compared with those obtained by the HPLC reference method and a good agreement was found between the two methods.

## 1. Introduction

In the last years, potentiometric methods with ion–selective electrodes have became important analytical tools in pharmaceutical analysis due to their advantages such as fast response times, good sensitivity and selectivity, low cost and simple design.

Among other classes of compounds used as active materials in the construction of potentiometric sensors, metalloporphyrins are one of the most promising. They are capable of binding the anions in a selective and reversible way conducting to membrane electrodes with selectivity significantly different from the Hofmeister selectivity pattern. There are several papers in which ion-selective electrodes porphyrin based are described [1–5]. Manganese(III) porphyrins are one of the most useful porphyrins used in the construction of polymeric membranes.

The first use of a manganese porphyrin as an active material was reported by Suzuki *et al.* [6] who employed manganese tetraphenylporphyrin (MnTPP-Cl) in a triiodide-selective electrode. The electrode shows a super–Nernstian response in a linear range 10^−5^ – 10^−3^ M triiodide (pH ranging from 2 to 9) with an anionic slope of 87 mV/dec.

Other manganese porphyrins were tested by Khorasani *et al*. [7] as ionophores in thiocyanate-selective sensors. The best results were obtained for the membrane sensor based on tetratolylporphine manganese(III) chloride (MnTTP-Cl) which has a wide linear range between 10^−7^ – 10^−1^ M (pH between 3 and 8). The sensor was applied for the determination of thiocyanate in urine samples.

In the pharmaceutical analysis manganese tetraphenylporphyrin (MnTPP-Cl) chloride was used for the first time in the construction of a penicillin G-selective sensor in a poly(vinyl) chloride matrix. The sensor has a linear response 2 × 10^−5^ – 10^−1^ M for penicillin-G, a slope of about 59 mV/dec and was used for the potentiometric analysis of penicillin-G in pharmaceutical products [8].

The same porphyrin was used by the authors [9] as an active material of a valproate–selective sensor. The obtained sensor has a slope of −60.8 mV/dec and a working concentration range 10^−1^ – 9.9 × 10^−6^ M with practical limit of detection of 4.8 × 10^−6^ M (pH from 4.5 to 8.4). The sensor was used for the determination of valproate in pharmaceutical preparations.

Saraswathyamma *et al*. [10] have tested the sensitivity and selectivity for paracetamol of carbon paste and polymeric liquid membrane electrodes incorporating manganese tetrakis (3,5-bis(*t*-butyl)phenyl) porphyrin chloride. The sensor based on carbon paste modified with this porphyrin displayed a better response toward paracetamol, having a detection limit of 3.9 × 10^−5^ M and a slope of −45.7 mV/dec. It was applied in the analytical determination of paracetamol in drugs.

**Figure 1.**
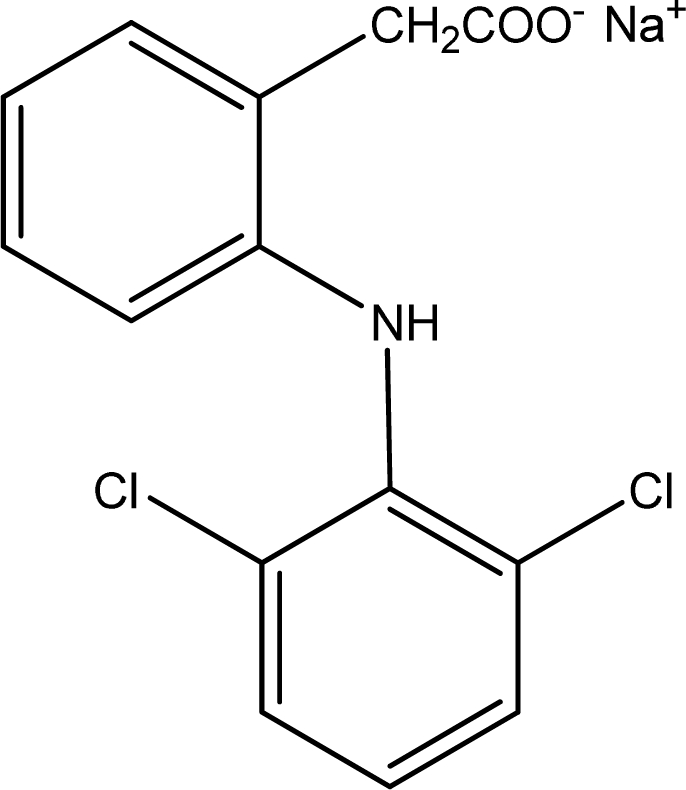
The chemical structure of sodium diclofenac.

Diclofenac (Figure 1) is a non–steroidal drug used in the treatment of rheumatoid arthritis, ankylosing spondylitis, osteoarthritis due to its analgesic, anti-inflammatory and antipyretic properties. A lot of analytical methods have been developed for the determination of diclofenac content in pharmaceutical form and biological fluids [11–13]. Among these, some ion-selective electrodes mainly based on ionic-pair complex of diclofenac with different cations were reported [14–19].

In the present paper we have used two manganese porphyrins as active materials for diclofenac-selective sensors. Two types of membranes, PVC and sol–gel, were evaluated. A comparison between the diclofenac–selective sensors reported in the literature and the sensor with the best potentiometric characteristics obtained by us is presented. In addition, we have used electrochemical impedance spectroscopy (EIS) to investigate the behavior of ion-selective membrane immobilized on the surface of a platinum electrode. An electrical equivalent circuit was used to interpret and fit the experimental results.

## 2. Experimental Section

### Reagents

The ionophores manganese(III) tetraphenylporphyrin chloride (MnTPP-Cl) (1) and manganese(III)-5,10,15,20-tetrakis(3-hydroxyphenyl)porphyrin chloride (2) were synthesized and purified according to literature data and characterized by UV–VIS, FT–IR and fluorescence spectroscopy [20]. PVC of high molecular weight, bis(2-ethylhexyl)sebacate (DOS), *o*-nitrophenyloctylether (NPOE), dioctylphtalate (DOP) were obtained from Merck and Aldrich. Tridodeocylmethylammonium chloride (TDMACl), sodium tetraphenylborate (NaTPB) and tetrahydrofuran (THF) of highest purity were available from Merck and Fluka and were used without further purification, except THF which was distilled before use. The sol–gel membranes were prepared using isobutyltriethoxysilane (IB–TES, Fluka), ethanol (Merck), hydrochloric acid (HCl, Merck), poly(ethylene glycol) (PEG200, Aldrich) and de-ionized water.

All aqueous solutions were prepared with salts of the highest available purity. The sample solutions for all potentiometric measurements consisted of sodium salts of the employed anions in 4-morpholinoethanesulfonic acid (MES), adjusted to pH 7.1 with NaOH (PVC membranes) and a 5.5 MES buffer for sol-gel membranes.

For the preparation of sodium diclofenac (Sigma) stock solution (1 × 10^−2^ M) a rigorous amount of solid was dissolved in the previously mentioned buffer. The standard solutions for all potentiometric measurements were prepared by diluting the stock solution with the pH adjusting buffer solution (pH = 7.1).

For the analysis of pharmaceutical samples by HPLC method, the mobile phase was a degasified and filtered mixture of methanol (Merck) and phosphate buffer (pH = 2.5) 70/30 v/v. The same mixture was used for the dilution of a 0.75 mg/mL diclofenac standard solution to obtain 0.075 mg/mL.

### Membrane preparation and electrode construction

The composition of the PVC membranes was: 1 wt% ionophore, 66 wt% plasticizer, 33 wt% PVC (plasticizer: PVC = 2:1). Various amounts of cationic (TDMACl) and anionic (NaTPB) additives (mol % relative to ionophore) were also used in some membranes. The electroactive material (5 mg) and different plasticizers (*o*-NPOE, DOP and DOS) (330 mg) were mixed together then 165 mg of PVC and the appropriate amount of THF (about 5 mL) were added and mixed to obtain a transparent solution. This solution was transferred onto a glass plate of 20 cm^2^ area, and the THF was allowed to evaporate at room temperature leaving a tough, flexible membrane embedded in a PVC matrix. Sol-gel membranes were prepared according to literature data [9] with a slight modification by using IB-TES as alkoxy reagent.

Prior to EMF measurements, the electrodes were conditioned for 2 h in a 0.01 M diclofenac solution. Potentiometric selectivity coefficients were determined according to the separate solution method [21] by using the experimental EMF values obtained for 10^−3^ M solutions of the test anions (at pH = 7.1) and a theoretical slope of 59.2 mV/dec diclofenac. The detection limit of the sensors was established at the point of intersection of the extrapolated linear mid-range and final low concentration level segments of the calibration plot.

For EIS measurements, a clean platinum electrode (area ≈1 cm^2^) was covered by the freshly prepared PVC–modified membrane (1% ionophore (I) + 66% DOP + 33% PVC + 20 mol % relative to ionophore NaTPB). The impedimetric measurements were focused on this specific membrane, due to the fact that it gave the best potentiometric response.

### Apparatus and electrodes

Potentiometric measurements were performed with the following galvanic cell: Ag/AgCl/KCl (sat)/sample/ion-selective membrane/Ag(Hg)/internal cable. All experiments were performed at ambient temperature (22 ± 2 °C). Potentials were measured using a Hanna Instruments HI223 pH/mV meter. Electrochemical impedance spectroscopy (EIS) measurements were performed using a Versastat 3 Potentiostat (V3 Studio software, Princeton Applied Research) connected with a three-electrode voltametric cell. A platinum electrode with large surface area (≈2 cm^2^) was employed as counter electrode while the reference was a standard calomel electrode (SCE). The impedance spectra were recorded over the frequency range 10 – 10^6^ Hz, by using a sinusoidal excitation signal (10 mV amplitude). The applied potential was 0 V *versus* calomel electrode. All experiments were carried out in quiescent solutions of MES buffer (pH 7.1) containing 10^−4^ M diclofenac. The EIS measurements were recorded both for the freshly prepared membrane and after it was conditioned for 24 hours in a MES buffer solution containing 10^−3^ M diclofenac. Data fitting was performed using ZSimWin software (Princeton Applied Research).

The chromatographic experiments were performed on a Waters ACQUITY BEH C18 column (2.1 × 100 mm, 1.7 μm) (Waters Corporation, Milford, MA, USA). The diclofenac standards were eluted with the following gradient program: 0–0.5 minute hold the initial condition, *i.e*., mobile phase A: 50% methanol in phosphate buffer, pH = 2.5; 100% methanol *versus* ammonium acetate (pH = 5) over 5 min followed by a hold at 100 % acetonitrile for 0.5 min before returning to original starting conditions over 0.1 min. The separations were performed on Waters ACQUITY Ultra Performance LC system with a mobile phase flow rate of 800 μL/min generating a column back-pressure up to 1.05 × 10^4^ psi.

### Sample preparation

Five commercial pharmaceutical formulations (tablets and ampoules) containing diclofenac were analyzed using the diclofenac–selective electrode. The liquid samples were prepared by measuring known volumes which were subsequently diluted in the buffer solution (pH 7.1). The solid samples (10 tables of each formulation) were weighted, powdered and homogenized. The amount corresponding to 7.5 mg of diclofenac was weighted and dissolved in 25 mL of buffer, set aside for 30 min and filtered. For the HPLC method, a homogenized aliquot of each sample was diluted with the methanol-water mixture to obtain a 0.075 mg/mL sodium diclofenac concentration. The solutions were filtered before use.

## 3. Results and Discussion

### 3.1. Potentiometric Measurements

**Figure 2.**
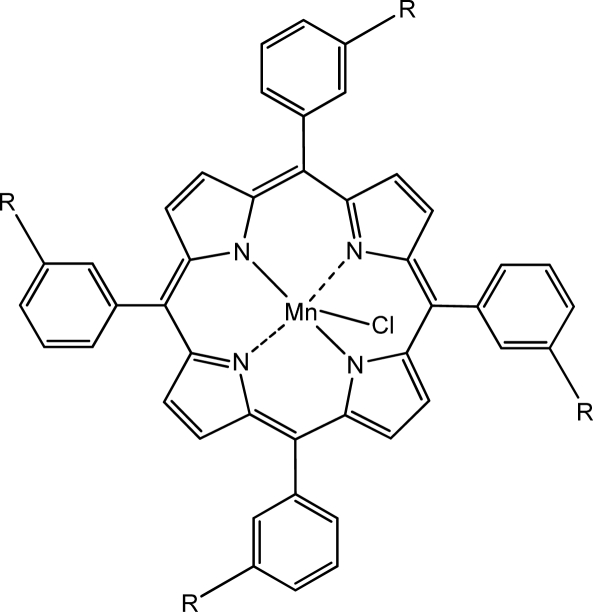
The chemical structure of the ionophores. Ionophore (1): manganese(III) tetraphenylporphyrin chloride, R = H; Ionophore (2): manganese(III)-tetrakis (3-hydroxyphenyl)porphyrin chloride, R = OH.

The chemical structure of the manganese(III) porphyrins used as ionophores is presented in Figure 2.

**Figure 3.**
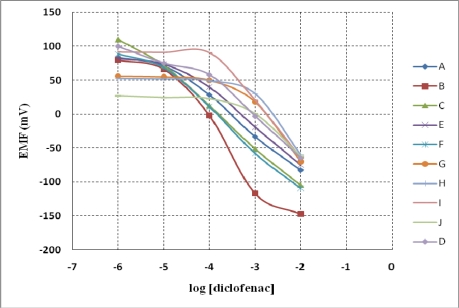
Potentiometric response to diclofenac of the obtained sensors.

In the case of manganese porphyrins both responses, charged and neutral, are possible due to the 3+ oxidation state of the central ion. This is the reason why membranes without additives and with anionic and cationic additives were prepared and their responses to the diclofenac anion were evaluated. The influence of three different plasticizers on the potentiometric response was also tested. The composition of the prepared membranes is presented in Table 1. The potentiometric response of the sensors to diclofenac is presented in Figure 3 and their working characteristics are presented in Table 2.

Analyzing the obtained results it can be seen that MnTPPCl-Cl is a better ionophore for diclofenac than the manganese hydroxyphenylporphyrin (ionophore 2) is. In the case of ionophore 2, for all the prepared sensors, incorporating anionic, cationic or without additive, the slope was supra–Nernstian with a narrow linear response. This may be due to a dimerization of the porphyrin in the membrane. For this reason the sensors based on manganese(III)-tetrakis(3-hydroxyphenyl)porphyrin chloride have no analytical applications and were not tested further.

**Figure 4.**
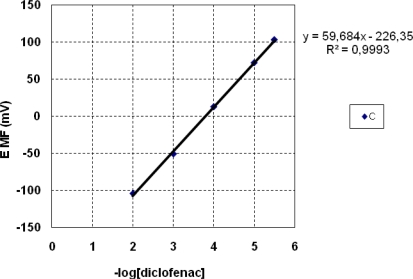
The potentiometric response of sensor C toward diclofenac.

For the MnTPP-Cl based sensors it results that the incorporation of cationic additive TDMACl (sensor B) conducts to sensors with worse potentiometric response than the membranes without additives, both in terms of linear concentration range and slope. The sensors having anionic additives in the membrane have an improved potentiometric answer (sensor C) with a linear range from 3 × 10^−6^ to 1 × 10^−2^ M diclofenac and a Nernstian slope of −59.7 mV/dec diclofenac (Figure 4).

The best results were obtained for the membrane having 20 mol% NaTPB. By increasing its concentration to 40 mol%, the sensor showed worse working characteristics (sensor D). It results that MnTPP-Cl acts as a charged carrier, the optimum composition of the membrane being obtained for membranes having 20 mol% NaTPB. Following, we have tested the influence of the plasticizer on the potentiometric answer. Three different plasticizers were used: DOP, DOS and *o*-NPOE and the potentiometric response of the resulting sensors was tested in the presence of diclofenac. In the case of DOS the sensor (E) exhibited a worse potentiometric answer; by using *o*-NPOE as plasticizer didn’t improve significantly the sensor behavior and the results were almost the same (sensor F).

The sol–gel membrane sensor had a linear potentiometric answer from 5 × 10^−4^ to 1 × 10^−2^ M with a near–Nernstian slope. The sensor characteristic was worse than that of PVC membrane sensor, so this sensor was not further tested.

One of the most important characteristic of ion-selective electrodes is selectivity, which describes the preference of the electrode for an interfering ion, relative to the primary one (diclofenac). Several organic and inorganic anions were used to test their interference with the primary anion. The potentiometric selectivity coefficients were calculated by the separate solution method with the interfering and primary anion at 10^−3^ M (for sensors C and F). A slight improvement of the selectivity coefficients was observed for the membrane plasticized with DOP (sensor C). This was considered by us the optimum membrane composition and the values of the selectivity coefficients are comparatively presented in Table 3 with those of diclofenac–selective sensors reported in the literature by now.

From the data presented in Table 3 it can be seen that the selectivity sequence for our sensor is different from the Hofmeister pattern (SCN^−^ > Sal^−^ > CH_3_COO^−^ > I^−^ > NO_2_^−^ > Cl^−^ > NO_3_^−^). This suggests a selective coordination of the anions with the central metal ion Mn(III). An important fact is that even for the most interfering anion, SCN^−^, the value of the selectivity coefficient is −1.36, showing that the sensor responded stronger to diclofenac than to thiocyanate, being substantially a diclofenac–selective one.

The influence of pH on the electrode potential was studied at a diclofenac concentration of 10^−3^ M. The pH was changed using small volumes of concentrated sulphuric acid or sodium hydroxide and recorded with a glass electrode.

**Figure 5.**
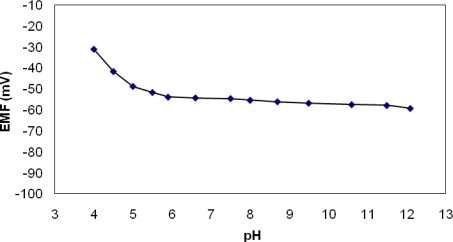
The pH influence on the potentiometric response.

As presented in Figure 5, there was only a slight variation of the potential value with the pH in the 5.5 – 11.5 pH range. It is already known that metalloporphyrins present a significant dependence on the pH. In the acidic region the electrode responded probably to H^+^ ions because the membranes contained carriers of amine nature (tetraphenylporphyrin) which may appear in the membrane surface layer as a result of partial demetallation of the metalloporphyrin complex during the membrane contact with the low pH test solution [22]. At high pH values diclofenac and HO^−^ ions are in competition to coordinate to the central metal ion (manganese) of the porphyrin. As in the case of iron porphyrins [19], it seems that diclofenac has a special ability to coordinate, so that the pH does not have any interference on the diclofenac response.

The potentiometric characteristics of the sensor C prepared by us are summarized in Table 4 and comparatively presented to those of the diclofenac-selective sensors reported in the literature.

### 3.2. Electrochemical Impedance Spectroscopy (EIS)

The impedance spectra were recorded for the PVC–modified membrane which gave the best potentiometric response (1% ionophore (1) + 66% DOP + 33% PVC + 20 mol% NaTPB relative to the ionophore).

**Figure 6.**
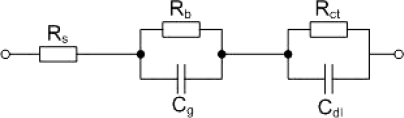
Equivalent circuit employed to fit the experimental EIS spectra.

The equivalent circuit (Figure 6) employed to fit the EIS experimental data contains the solution resistance (R_s_) and two parallel RC pairs: R_b_, C_g_ respectively R_ct_, C_dl_.

**Figure 7.**
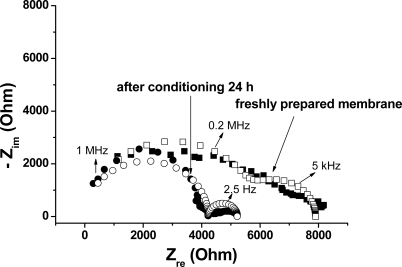
Nyquist plot for the freshly prepared membrane and for the conditioned membrane; the open symbols represent the fit of the experimental data, based on the electrical circuit presented in Figure 6.

The solution resistance R_s_ is generally low (hundreds of Ohm) and depends on the types of ions and their concentration, solution temperature as well as the geometry of the area in which the current is carried. The first RC pair characterizes the bulk resistance of PVC membrane (R_b_) and its geometrical capacitance (C_g_) and appears in the EIS spectrum as a depressed semicircle (high frequency range, Figure 7). The changes in the high frequency resistance are related with the state of the membrane: freshly prepared or conditioned. We can expect that the resistance of the freshly prepared membrane is considerably larger than that of the conditioned membrane.

The second RC pair characterizes the interfacial contributions of charge–transfer resistance (R_ct_) and double–layer capacitance (C_dl_) and appears as a low–frequency semicircle (Figure 6). The measurement of R_ct_ in such membranes may give information on the reaction kinetics at the membrane/solution interface [23].

The total impedance Z_tot_ of the equivalent circuit is given by Equation (1). The real (Z_re_) and imaginary (Z_im_) part are expressed by Equation (2) respectively 3, while the absolute impedance (/Z/) is given by Equation (4). Equation (1) has been used for fitting the experimental results.

(1) $$Z_{\mathrm{tot}}=R_{s}+\frac{R_{b}}{1+\omega^{2}C_{g}^{2}R_{b}^{2}}+\frac{R_{\mathrm{ct}}}{1+\omega^{2}C_{\mathrm{dl}}^{2}R_{\mathrm{ct}}^{2}}-j\left(\frac{\omega C_{g}R_{b}^{2}}{1+\omega^{2}C_{g}^{2}R_{b}^{2}}+\frac{\omega C_{\mathrm{dl}}R_{\mathrm{ct}}^{2}}{1+\omega^{2}C_{\mathrm{dl}}^{2}R_{\mathrm{ct}}^{2}}\right)$$

(2) $$Z_{\mathrm{re}}=R_{s}+\frac{R_{b}}{1+\omega^{2}C_{g}^{2}R_{b}^{2}}+\frac{R_{\mathrm{ct}}}{1+\omega^{2}C_{\mathrm{dl}}^{2}R_{\mathrm{ct}}^{2}}$$

(3) $$Z_{\mathrm{im}}=\frac{\omega C_{g}R_{b}^{2}}{1+\omega^{2}C_{g}^{2}R_{b}^{2}}+\frac{\omega C_{\mathrm{dl}}R_{\mathrm{ct}}^{2}}{1+\omega^{2}C_{\mathrm{dl}}^{2}R_{\mathrm{ct}}^{2}}$$

(4) $$|Z|=\sqrt{{\left(Z_{\mathrm{re}}\right)}^{2}+{\left(Z_{\mathrm{im}}\right)}^{2}}$$

The Nyquist plot of the EIS data for the modified electrode (freshly prepared and after conditioning 24 hours in 10^−3^ M diclofenac) shows two semicircles which arise from the coupling of R_b_, C_g_ respectively R_ct_, C_dl_ (Figure 7). The bulk resistance R_b_ is very high (around 6 kΩ) for the freshly prepared membrane and significantly decreased after the membrane was conditioned for 24 hours (≈4 kΩ).

At the lowest frequencies, the contribution from Warburg impedance due to diffusion of electroactive species to the reaction interface does not appear (straight line, at an angle of 45 degrees). Warburg impedance was reported before for PVC membranes, only if the concentration of carrier and mobile site species were approximately equal to each other [24,25].

The two parallel RC pairs are characterized by a characteristic time constant, τ(τ = RC). Using the values obtained for the fit parameters (R_b_C_g_ respectively R_ct_C_dl_) we have determined the time constant for the two circuits. In the case of freshly prepared membrane, the high-frequency semicircle has a time constant of ≈3.74 × 10^−7^s (R_b_ = 5,500 Ohm; C_g_ = 6.8 × 10^−11^ F) which is close to that of conditioned membrane ≈4.66 × 10^−7^s (R_b_ = 4,200 Ohm; C_g_ = 1.11 × 1^−10^ F). This can be explained by the fact that the conditioned membrane has a lower bulk resistance, but at the same time a higher capacitance than the dry membrane (due to accumulation of diclofenac ions inside the membrane, which leads to an increase of the total charge).

The low–frequency semicircle which corresponds to the coupling of charge-transfer resistance (R_ct_) and double layer capacitance (C_dl_) has a time constant of ≈1.21 × 10^−5^ s (R_ct_ = 2,250 Ohm; C_dl_ = 5.42 × 10^−9^ F) for the freshly prepared membrane. It considerably increases after conditioning the membrane to ≈1.07 × 10^−2^ s (R_ct_ = 1,000 Ohm; C_dl_ = 1.07 × 10^−5^ F).

In order to be used as ion-selective sensors, the membrane has to have a reduced resistance (not to hinder the movements of ions inside/out and the transfer of electrons) as well as a high capacitance (membranes with higher capacitance are more stable in time [26]). From the ISE measurements it appears that the conditioned membrane has better characteristics for its use in potentiometric determination of diclofenac, as it offers a considerably lower resistance. In addition, its capacitance is order of magnitude higher than that of dry membrane, due to accumulation of diclofenac ions inside it.

**Figure 8.**
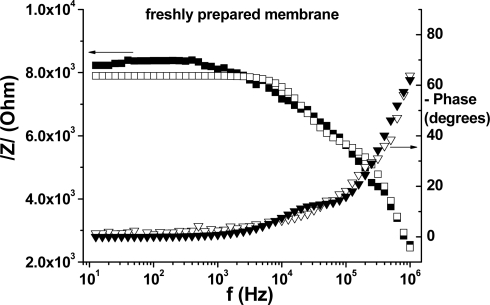
Bode plot for the freshly prepared membrane; the open symbols represent the fit of the experimental data, based on the electrical circuit presented in Figure 6.

**Figure 9.**
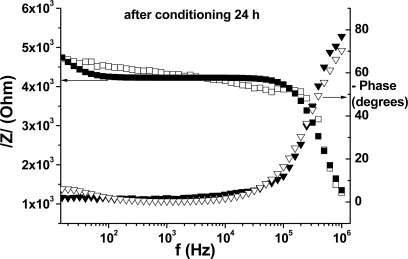
Bode plot for the conditioned membrane; the open symbols represent the fit of the experimental data, based on the electrical circuit presented in Figure 6.

Another useful representation of the EIS data is the Bode plot, which is shown in Figures 8 and 9 (for freshly prepared respectively conditioned membrane). This representation allows us to examine the absolute impedance, /Z/, and the phase angle as a function of frequency. When the phase angle between the applied voltage and the current is zero, then a pure resistance is present.

When a phase angle of 90° is measured between the voltage and current at the same frequency, a pure capacitance is present. Angles between these values mean that the circuit consists in a combination of resistors and capacitors. As can be seen in these Figures, at very high frequencies (10^6^ Hz) the membrane behavior is mainly capacitive, with the phase angle close to 70 degrees. At low and intermediate frequencies (10−10^4^ Hz) the membrane behavior is mainly resistive, with the phase varying between 0 and 10 degrees. Within this frequencies range the absolute impedance, /Z/, is very high for the fresh membrane (around 8.3 kΩ) and considerably decreased after conditioning 24 h (≈4.2 kΩ).

### 3.3. Analytical Applications

Sensor C having the optimum composition of the membrane was used for the determination of diclofenac in solid (tablets) and liquid (injectables) drugs. The samples were prepared as previously described and the diclofeanc assay was carried out by direct potentiometry. In Table 5 are presented the results (average of three measurements) obtained by potentiometry and HPLC (used as the reference method). The results are in good agreement.

## 4. Conclusions

A new diclofenac–selective sensor based on manganese(III) porphyrins was developed by us. The interaction mechanism in the case of MnTPP-Cl at pH 7.1 is a charged one; the addition of NaTPB to membrane composition led to the improvement of potentiometric answer. The membrane having the optimum composition was characterized by EIS. The sensor has the best sensitivity from all the diclofenac selective sensors presented in the literature, with very good values for the selectivity coefficients too. It can be used in the pH range 5.5 – 11.5.

The sensor was also used for determination of diclofenac from pharmaceutical preparations and the results were compared with those obtained by HPLC method, employed as reference. The good agreement between the two methods makes the diclofenac–selective sensor a useful alternative for the selective determination of diclofenac in drugs.

## Figures and Tables

**Table 1.** Membrane composition (% w/w) of the diclofenac–selective sensors.

**Sensor**	**Ionophore (1)**	**Ionophore (2)**	**Plasticizer**			**PVC**	NaTPB^*^	TDMACl^*^
			**DOP**	**DOS**	***o*-NPOE**			

A	1	-	66	-	-	33	-	-
B	1	-	66	-	-	33	-	20
C	1	-	66	-	-	33	20	-
D	1	-	66	-	-	33	40	-
E	1	-	-	66	-	33	20	-
F	1	-	-	-	66	33	20	-
G	-	1	66	-	-	33	-	-
H	-	1	66	-	-	33	20	-
I	-	1	66	-	-	33	-	20
J	Sol–gel	-						

* mol % relative to ionophore

**Table 2.** General working characteristics of the constructed sensors.

**Electrode**	**Linear concentration range (M)**	**Detection limit (μM)**	**Slope (mV/dec)**

**A**	1 × 10^−5^–1 × 10^−2^	5	−52.3 ± 0.8
**B**	1 × 10^−5^–5 × 10^−2^	5	−80.1 ± 1.2
**C**	3 × 10^−6^–1 × 10^−2^	1.5	−59.7 ± 0.6
**D**	5 × 10^−5^–1 × 10^−2^	30	−65.5 ± 0.8
**E**	1 × 10^−5^–1 × 10^−2^	8	−50.4 ± 0.5
**F**	5 × 10^−6^–1 × 10^−2^	3	−60.2 ± 0.9
**G**	5 × 10^−4^–1 × 10^−2^	400	−81.1 ± 1.1
**H**	3 × 10^−4^–1 × 10^−2^	200	−85.3 ± 1.2
**I**	1 × 10^−4^–1 × 10^−2^	150	−77.4 ± 0.9
**J**	5 × 10^4^–1 × 10^−2^	350	−62.3 ± 0.7

**Table 3.** Selectivity coefficients $\text{log}\;K_{\text{diclofenac},X}^{\text{pot}.}$.

**Interfering ion (X^−^)**	**Present research**	**Ref.[14]**	**Ref.[15]**	**Ref.[16]**	**Ref. [17]**	**Ref.[18]**	**Ref [19]**

Acetate	−2.00 ± 0.02	-	-	-	−2.9	-	−2.45
Phosphate	−4.15 ± 0.05	-	−3.8	-	-	-	−3.50
Nitrite	−2.31 ± 0.03	-	−3.2	-	-	-	−2.05
Benzoate	−2.38 ± 0.02	−2.40	−3.3	-		−5.0	−2.75
Tartrate	−2.43 ± 0.03	-	−3.6	-	−2.1	-	−4.40
Borate	−3.64 ± 0.04	-	-	-	-	-	−2.40
Thiocyanate	−1.36 ± 0.01	-	−3.5	-	−1.42	-	−0.90
Glycine	−2.43 ± 0.02	-	-	−2.6	-	−5.1	-
Iodide	−2.29 ± 0.03	-	−2.9	-	-	−4.3	-
Metabisulphite	−3.90 ± 0.06	-	-	-	-	-	−2.50
Chloride	−2.54 ± 0.01	-	−2.3	−2.6	−0.36	−4.7	−2.60
Lactate	−2.48 ± 0.02	-	-	-	-	-	-
Citrate	−4.14 ± 0.07	-	−3.8	-	-	-	−3.45
Nitrate	−2.58 ± 0.04	−2.50	−2.0	−2.3	-	−4.5	−2.30
Glucose	−2.52 ± 0.01	-	−3.2	−2.8	-	−4.9	-
Salicylate	−1.90 ± 0.01	−1.00	−2.7	-	−2.0	−4.7	−0.95
Sulphate	−4.18 ± 0.05	-	−3.0	-	−3.9	-	−3.70

**Table 4.** Comparative potentiometric characteristics of diclofenac–selective sensors.

**Reference no.**	**Linear concentration range (M)**	**Detection limit (M)**	**pH range**	**Slope (mV/dec)**
[14]	5 × 10^−5^–5×10^−2^	2.5 × 10^−5^	6−11	−59 ± 1
[15]	9 × 10^−6^–1×10^−2^	5.4 × 10^−6^	5.5−9	−61 ± 1
[16]	1 × 10^−5^–1×10^−2^	4.0 × 10^−6^	6–9	−59 ± 1
[17]	5 × 10^−5^–1×10^−2^	3.2 × 10^−5^	6.5–9	−58.1 ± 0.8
[18]	1 × 10^−5^–5×10^−2^	5.0 × 10^−6^	2.4–7.5; 8.5−11.6	−60 ± 1
[19]	5 × 10^−5^–1×10^−2^	2.0 × 10^−5^	8.8−12.8	−59.8 ± 0.3
Present research	3 × 10^−6^–1×10^−2^	1.5 × 10^−6^	5.5−11.5	−59.7 ± 0.6

**Table 5.** Determination of diclofenac in pharmaceutical formulations.

**Sample**	**Label amount (mg)**	**Found by potentiometry (mg)**	**Found by HPLC (mg)**	**Relative error (%)**
Diclofenac	50.0/tablet	49.6 ± 0.7	49.2 ± 0.4	+0.8
Diclotard	100.0/tablet	109.6 ± 1.3	108.4 ± 1.2	+1.1
Refen retard	100.0/tablet	105.9 ± 1.1	107.2 ± 0.9	−1.2
Voltaren	75.0/ampoule of 3 mL	79.2 ± 0.9	78.3 ± 0.8	+1.1
Refen	75.0/ampoule of 3 mL	74.0 ± 0.8	73.3 ± 0.6	+0.9
